# Bioactive compounds of pequi pulp and oil extracts modulate antioxidant activity and antiproliferative activity in cocultured blood mononuclear cells and breast cancer cells

**DOI:** 10.29219/fnr.v66.8282

**Published:** 2022-01-27

**Authors:** Renata Moraes Brito, Milene Teixeira Barcia, Carla Andressa Almeida Farias, Rui Carlos Zambiazi, Patrícia Gelli Feres de Marchi, Mahmi Fujimori, Adenilda Cristina Honorio-França, Eduardo Luzia França, Paula Becker Pertuzatti

**Affiliations:** 1 Programa de Pós-Graduação em Imunologia e Parasitologia Básicas e Aplicadas, Universidade Federal de Mato Grosso, Campus Universitário do Araguaia, Barra do Garças, Brazil; 2 Programa de Pós-Graduação em Ciência e Tecnologia de Alimentos, Universidade Federal de Santa Maria, Santa Maria, Brazil; 3 Centro de Ciências Químicas, Farmacêuticas e de Alimentos, Universidade Federal de Pelotas, Pelotas, Brazil

**Keywords:** immunomodulation, cancer, carotenoids, phytosterols, tocopherols

## Abstract

**Background:**

Pequi (*Caryocar brasiliense* Camb.) is a fruit from Brazilian Cerrado rich in bioactive compounds, such as phytosterols and tocopherols, which can modulate the death of cancer cells.

**Objective:**

In the present study, the main bioactive compounds of hydrophilic and lipophilic extracts of pequi oil and pulp were identified and were verified if they exert modulatory effects on oxidative stress of mononuclear cells cocultured with MCF-7 breast cancer cells.

**Study design:**

Identification and quantification of the main compounds and classes of bioactive compounds in pequi pulp and oil, hydrophilic, and lipophilic extracts were performed using spectroscopy and liquid chromatographic methods, while the beneficial effects, such as antioxidant capacity *in vitro*, were determined using methods based on single electron transfer reaction or hydrogen atom transfer, while for antioxidant and antiproliferative activities *ex vivo*, 20 healthy volunteers were recruited. Human peripheral blood mononuclear cells (MN) were collected, and cellular viability assay by MTT *(3-(4,5-dimethylthiazol-2-yl)-2,5 diphenyltetrazolium bromide)*, superoxide anion evaluation, and *CuZn-superoxide dismutase determination (CuZn-SOD)* in MN cells, MCF-7 cells, and coculture of MN cells and MCF-7 cells in the presence and absence of pequi pulp or oil hydrophilic and lipophilic extracts were performed.

**Results:**

In the hydrophilic extract, the pequi pulp presented the highest phenolic content, while in the oil lipophilic extract, it had the highest content of carotenoids. The main phytosterol in pequi oil was β-sitosterol (10.22 mg/g), and the main tocopherol was γ-tocopherol (26.24 μg/g sample). The extracts that had highest content of bioactive compounds stimulated blood mononuclear cells and also improved SOD activity. By evaluating the extracts against MCF-7 cells and coculture, they showed cytotoxic activity.

**Conclusion:**

The results support the anticarcinogenic activity of pequi extracts, in which the pequi pulp hydrophilic extracts presented better immunomodulatory potential.

## Popular scientific summary

- This is the first study reporting the content of phytosterols and tocopherols in pequi pulp oil, which shows the important potential stimulating the human peripheral blood mononuclear cells and was cytotoxic to MCF-7 breast cancer cells and coculture of MN cells and MCF-7 cells, especially the pequi pulp hydrophilic extract.
- These results may contribute to future work, regarding the use of these extracts for breast cancer prophylactic measures.

Cancer incidence and mortality are growing rapidly worldwide, and the breast cancer is the most commonly diagnosed cancer and the leading cause of cancer death among females (1). The use of new drugs and alternative treatments that act against cancer due to a modulation of the immune system, which are obtained mainly from natural sources, such as fruit, is interesting due to the reduction of side effects in the treatment. To induce, tumor cell death, specifically, is essential once, normal cells should not be affected, or at least this effect should be minimized, being the peripheral blood an interesting source for assessing the functional competence of subgroups of immune cells (2).

Bioactive compounds of Brazilian Cerrado plants and its antioxidant activity are associated with antiproliferative action in breast cancer cells (3–5). Pequi (*Caryocar brasiliense* Camb.) is an oleaginous fruit typically from the Brazilian Cerrado, composed by exocarp (an outer green bark), mesocarp (the inner part of the bark), endocarp (an orange or yellow pulp), thorn, and seed layer, which has bioactive compounds with antioxidant properties such as carotenoids, mainly β-carotene and lycopene, and ascorbic acid (6, 7). Pequi almond oil is rich in phytosterols and tocopherols (8, 9). However, the biological properties of pequi oil can change according to the oil source (pulp or almond)^.^ (10), and to our knowledge, there are no studies reporting the content of these compounds in pequi pulp oil. Beyond their cardiovascular applications and strong antioxidant activity, phytosterols and tocopherols, respectively, may also posses anticancer properties, as has been demonstrated by several studies (11–14).

The aim of this study was to identify the main compounds of pequi pulp and oil hydrophilic and lipophilic and to verify the modulatory effects of them on oxidative stress of human peripheral blood mononuclear (MN) cells cocultured with MCF-7 breast cancer cells.

## Materials and methods

### Samples

The mature pequi, around 1.7 kg of pulp, belonging to the 2018 crop, was obtained in the city of Barra do Garças, Mato Grosso, Brazil (geographical coordinates 15° 53’ 24” S, 52° 15’ 24” W). After harvesting the fruits, they were selected, sanitized, packed in polyethylene plastic bags, and stored at –18°C throughout the experiment.

The oil from the pequi pulp (endocarp) was extracted with petroleum ether using the Goldfish (Tecnal, model TE – 044, São Paulo-Brazil) extractor apparatus for 5 h 20 min at 90°C.

To obtain the pequi pulp or oil lipophilic extract, the extraction was carried out according to the adapted method of Rodriguez-Amaya, which differed in relation to the sample amount (0.5 g of pulp and 1 g of oil) (15). The sample was then added by 20 mL of ice-cold acetone and continued stirring for 10 min on Ultrasound (SoniClean 6), followed by filtration. This procedure was repeated three times for the exhaustive removal of the compounds. Then, a partition was made with petroleum ether and distilled water, and the ethereal extract was used for analyses – the extracts were analyzed shortly after extraction to avoid any alteration of the analyte.

The hydrophilic extract prepared from the pequi pulp was prepared adding 25 mL of the extractive solution composed of methyl alcohol:water:formic acid (50:48.5:1.5, v/v/v) in 5.0 g of pequi pulp, and then the solution was stirred for 15 min under ultrasound. Filtration was then carried out – the residue was reextracted, and the filtrates were combined.

The pequi oil hydrophilic extract was prepared according to the modified method of Nakbi et al. (16). The extracts were analyzed immediately to avoid decomposition of the analytes.

### Bioactive compounds characterization

The performance to determine the total phenolic compounds (TPC) was according to the colorimetric method adapted from Singleton et al., using the colorimetric assay based on the Folin Ciocalteau reagent (17). The spectrophotometer (Kasuaki UV-VIS) absorbance reading was carried out at 740 nm. A standard curve of gallic acid (0.01–0.08 mg/mL) was drawn for quantification.

The colorimetric method, adapted from Arvouet-Grand et al., determined the total flavonoid content based on the reaction with aluminum chloride (18). A standard quercetin curve (0–40 mg/L) (90% of purity; Sigma-Aldrich Chemical Company, Steinheim, Germany) was elaborated for quantification.

For the identification and quantification of phenolic compounds, a 20 μL of the pequi oil hydrophilic extract was injected into the high-performance liquid chromatography (HPLC-DAD, Shimadzu). The chromatographic method used for the separation was based on the study of Quatrin et al., in which a Hypersil Gold C-18 reverse phase column (5 μm of particle, 150 mm, 4.6 mm) was used with an oven temperature of 38°C and two mobile phases, composed of 5% methyl alcohol, 0.1% formic acid, and milli-Q water (mobile phase A) and acetonitrile acidified with 0.1% formic acid (mobile phase B) (19). Calibration curves of epicatechin (280 nm, flavanols) (>97% of purity; ChromaDex, California, USA) and chlorogenic acid (320 nm, hydroxycinnamic acids) (98.05% of purity; Chem-Impex International, Illinois, USA) were used to quantify the different phenolic classes.

For the determination of total carotenoids, the lipophilic extracts were read directly in a spectrophotometer at 450 nm, according to the method described by Rodriguez-Amaya (15).

For the tocopherols analysis, a 0.2 g of pequi oil was diluted in 5 mL of isopropyl alcohol and centrifuged at 9,000 rpm for 6 min (NT800 microcentrifuge, Nova Técnica – São Paulo, Brazil). After preparation of the extract, a 10 μL was injected in HPLC (Shimadzu), equipped with automatic injector and fluorescence detector, with excitation and emission wavelengths, 290 nm and 330 nm, respectively. The separation was carried out on a reverse phase RP-18 column (5 μm, 4.6 mm × 150 mm) with octadecyl stationary phase, operating at 25°C with a flow of 1.0 mL/min. The separation was performed according to Pertuzatti et al. using a gradient elution system, using methyl alcohol, acetonitrile, and isopropyl alcohol as mobile phases at a ratio of 40:50:10 to 30:65:5 (v/v/v) in the first 10 min, followed by a linear gradient of 50:40: 10–12 min and maintaining this ratio until the end of the run (15 min) (20). For the quantification of α-tocopherol, (δ+β)-tocopherol, and γ-tocopherol, an external standard curve was used, prepared with the corresponding chromatographic standards, and the total tocopherol content was expressed in μg/g sample, determined by the sum of the individual tocopherols. The standards of γ- and δ-tocopherols were obtained, with 96 and 90% of purity, respectively; α-tocopherol was obtained from Sigma Aldrich (Steinheim, Germany) with 99% of purity.

For the determination of phytosterols, about 3 g of oil was weighed; subsequently, it was added 25 mL of potassium hydroxide equivalent-gram/L in ethyl alcohol (m/v), and the sample was stirred every 15 min for 1 h and then maintained resting for 18 h, for complete saponification. After saponification, a partition was performed, and the ether phase was rotated at 40°C. The extract was resuspended in 5 mL of methyl alcohol:ethyl alcohol (1:1, v/v) and centrifuged at 9,000 rpm for 6 min. Then, a 25 μL was injected into the same chromatograph described for the tocopherols method, using a 250 nm ultraviolet detector and isocratic elution of methyl alcohol for 20 min with 0.7 mL/min of flow. The identification of the compounds was performed by comparing the retention time according to the standard campesterol, stigmasterol, and β-sitosterol (Sigma, St. Louis, USA) phytosterols curves, and the total phytosterols content was expressed in mg/g of sample.

### *Determination of* in vitro *antioxidant capacity*

#### DPPH free radical capture

The method of Brand-Williams et al. was used to determine the DPPH free radical capture (21). In tubes protected from light, a 100 μL of the sample extract (hydrophilic or lipophilic extract) was added to 3.9 mL of the use solution of the free radical DPPH, and after 30 min of reaction, the absorbance was measured at 517 nm in a spectrophotometer (Kasuaki UV-VIS) and compared with a standard Trolox curve ((±)-6-hidroxy-2,5,7,8-tetramethylchroman-2-carboxylic acid (Trolox^®^, 97%; Sigma-Aldrich Chemical Company, Steinheim, Germany)).

#### Free radical ABTS capture (2,2’-azinobis (3-ethyl-benzothiazoline-6-sulphonate)

The method was performed as described by Re et al. (22). a 30 μL of the samples of hydrophilic or lipophilic extract was added in a test tube, together with a 3 mL of diluted ABTS solution (ABTS, ≥ 98%; Sigma-Aldrich Chemical Company, Steinheim, Germany). After incubation for 25 min at 30°C, absorbance was read at 734 nm in a spectrophotometer (Kasuaki UV-VIS) and compared with a standard Trolox curve.

#### Ferric‑Reducing Antioxidant Power (FRAP)

The FRAP analysis was performed according to Benzie and Strain (23). Each tube was added 2,400 μL of FRAP solution, 240 μL of distilled water, and 80 μL of hydrophilic extract. After mixing, it was placed in a water bath for 15 min at 37°C, and the absorbance reading was made at 593 nm in a spectrophotometer (Kasuaki UV-VIS) and compared with a standard Trolox curve.

#### Radical Oxygen Absorption Capacity (ORAC)

For the analysis of ORAC, the method of Ou et al. was used, where a 25 μL of hydrophilic extract, 50 times diluted, was added to the microplates together with 150 μL of fluorescein (81 nmol/L) and 25 μL of 2,2’-azobis (2-amidino-propane) dihydrochloride (AAPH) (152 mmol/L); AAPH and fluorescein disodium were purchased from Sigma-Aldrich Chemical Company (Steinheim, Germany). The plates were incubated in a microplate reader (Hidex Sense) at 37°C, and readings were taken every 1 min for a period of 90 min, using the fluorescence detector with 485 nm excitation wavelength and emission at 528 nm (24). A standard Trolox curve was drawn on each test microplate.

### Bioassays

To bioassays, the four extracts (oil lipophilic extract, oil hydrophilic extract, pulp lipophilic extract, and pulp hydrophilic extract) were subjected to a rotary evaporator at 45°C, and the lipophilic extract was diluted in dimethylsulfoxide; both fractions were elevated in water.

To perform the separation of blood mononuclear cells, a 10 mL of human peripheral blood from clinically healthy volunteers (*n* = 20) with an average age of 30 years was collected in Vacutainer ethylenediamine tetraacetic acid (EDTA) tubes (Beckton Dickinson, Franklin Lakes, NJ, USA^®^). The volunteers signed an informed consent form before entering the study, which was approved by the local ethics committee of the Federal University of Mato Grosso (Protocol Number CAAE: 2715319.0.0000.5587).

Cell populations were then separated by Ficoll-Paque density gradient (Pharmacia Upsala-Sweden) through centrifugation for 40 min at 1,600 rpm at room temperature (25°C). The enriched ring of human peripheral blood mononuclear cells (MN), formed during centrifugation, was withdrawn with the aid of a Pasteur pipette and washed twice with 4 mL of phosphate buffered saline (PBS). Then, the supernatant was removed, and a 1 mL of PBS was added.

Cells were counted in the Neubauer’s chamber, and the cell concentrations were adjusted to 2 × 10^6^ cells/mL culture medium.

Subconfluent monolayers (80%) of MCF-7 breast cancer cells were treated with trypsin (Sigma, St. Louis, USA) at a concentration adjusted to 5 × 10^4^ cells/mL. The trypsinized cells were preincubated for 24 h with or without pequi pulp or oil hydrophilic or lipophilic extract (100 pg/mL, 100 ng/mL, and 100 mg/mL). The cells were then resuspended in RPMI-1640 medium supplemented with 10% fetal bovine serum (FBS) (Sigma, St. Louis, MO, USA), penicillin (20 U/mL), and streptomycin (20 µg/mL) (Sigma, St. Louis, MO, USA) at 37°C for 24 h in a humidified atmosphere containing 5% CO_2_.

Coculture analyses were performed following the same protocols described above for MCF-7 cells together with MN cells.

Cellular viability assay by MTT (3-(4,5-dimethylthiazol-2-yl)-2,5 diphenyltetrazolium bromide) was also performed.

The evaluation of cytotoxic effects of pequi oil and pulp hydrophilic and lipophilic extracts used the MTT colorimetric method with blood mononuclear cells, MCF-7 cells, and coculture of MN cells and MCF-7 cells, whose base is on the reduction of the tetrazolium ring of MTT by mitochondrial succinate dehydrogenases, yielding a blue formazan product that can be measured spectrophotometrically (25). The amount of formazan produced is proportional to the number of viable cells (26).

Initially, the extract was added a 100 μL of the MN culture, MCF-7 cells, and coculture of MN cells and MCF-7 cells to a 96-well plate, and then a 100 μL of RPMI-1640 medium (without phenol with 5 g/100 g FBS) was added and treated with different concentrations (100 pg/mL, 100 ng/mL, and 100 mg/mL) of the pequi oil and pulp extracts. Controls (cells and culture medium) were also elaborated and then incubated in a drying oven at 26°C for 24 h. After this treatment, a 20 μL of MTT (5 mg/mL) was added to each well, and the plates were incubated at 37°C with 5% CO_2_ for 3 h. The metabolically active cells reduced the MTT to the blue formazan crystals, which were dissolved in 50 μL solution of sodium dodecyl sulfate (SDS), leaving the plate under stirring for approximately 20 min for the dissolution of the crystals and obtaining a translucent solution. The absorbance was determined at 492 nm using a microplate reader (Thermo Plate, TP Reader).

#### Superoxide anion (O_2_^●-^)

The evaluation of superoxide anion (O_2_^●-^) release by human peripheral blood mononuclear cells, MCF-7 cells, and coculture of MN cells and MCF-7 cells in the presence and absence of pequi pulp or oil hydrophilic and lipophilic extracts was performed by incubating the extracts for 24 h and then centrifuging at 1,500 rpm for 10 min. The cell and stimulus suspension were resuspended in PBS containing 2.6 mmol/L of CaCl_2_ (m/v), 2 mmol/L of MgCl_2_, and cytochrome C (m/v). They were then incubated at 37°C for 24 h, in the absence of light and with CO_2_ control. The reading was done using a spectrophotometer (Thermo Plate TP-Reader) with a 540 nm filter. The calculation of superoxide anion concentration was according to the method adapted from Pick and Mizel (27).

#### CuZn-superoxide dismutase determination (CuZn-SOD)

For the antioxidant activity assay based on the dismutation of superoxide dismutase (SOD) in human peripheral blood mononuclear cells, MCF-7 cells, and coculture of MN cells and MCF-7 cells, supernatants treated or not treated with the pequi pulp and oil extracts using the NitroBlue tetrazolium (NBT) reduction method were pipetted in different tubes, 500 μL of the cell suspension supernatant and pequi pulp or oil extract. Then, in each tube, the following were added in order: 500 μL of chloroform-ethyl alcohol mixture (1:1, v/v), 500 μL of the NBT reactive mixture and EDTA (1:1.5, v/v), and 2 mL of the hydroxylamine buffer solution. For the calibration of the apparatus, the reactive mixture of NBT, EDTA, and 2 mL of hydroxylamine buffer was used.

Results were expressed in the international unit of measurement (UI) of CuZn-SOD.

### Statistical analyses

The results were submitted to one-way analysis of variance (one-way ANOVA). The Tukey multiple range test was used to determine the differences in mean values between different extracts (*P* ˂ 0.05, for characterization of extracts, and *P* ˂ 0.01, for the bioassays). Correlations among obtained data were calculated using the Pearson’s correlation coefficient (r). All analyses were made in triplicates, and the results were given as means ± standard deviation.

## Results

### Bioactive compounds characterization

The bioactive compounds present in the pequi pulp and oil extracts were evaluated, and the content of total phenolics and total flavonoids present in the hydrophilic extract is shown in Table 1, as well as the content of flavanols and phenolic acids obtained by chromatography.

**Table 1:** Total phenolic compounds (TPC), total flavonoids, flavanols, phenolic acids, carotenoids, and antioxidant capacity by the ABTS (2,2’-azinobis (3-ethyl-benzothiazoline-6-sulphonate), DPPH (2,2-diphenyl-1-picrylhidrazyl), Ferric‑Reducing Antioxidant Power (FRAP), and Radical Oxygen Absorption Capacity (ORAC) methods

Hydrophilic extract	Pequi pulp	Pequi oil
TPC (mg gallic acid/100 g)	54.60 ± 1.1^a^	4.40 ± 0.3^b^
Total flavonoids (mg quercetin/100 g)	13.20 ± 0.9^a^	1.10 ± 0.02^b^
Flavanols (mg epicatechin/100 g)	48.02 ± 0.02^a^	13.06 ± 1.92^b^
Phenolic acids (mg chlorogenic acid/100 g)	ND^b^	1.27 ± 0.12^a^
ABTS (µmol trolox/100 g)	408.03 ± 32.7^a^	69.35 ± 3.0^b^
DPPH (µmol trolox/100 g)	317.25 ± 25.5^a^	26.72 ± 2.5^b^
FRAP (µmol trolox/100 g)	74.31 ± 1.97^a^	1.15 ± 0.1^b^
ORAC (µmol trolox/100 g)	4,216.22 ± 70.2^a^	787.61 ± 51.1^b^
Lipophilic extract	Pequi pulp	Pequi oil
Carotenoids (μg β-carotene/g)	137.60 ± 13.1^b^	549.84 ± 8.9^a^
ABTS (µmol trolox/100 g)	671.05 ± 62.1^a^	772.74 ± 10.4^a^
DPPH (µmol trolox/100 g)	973.40 ± 90.5^b^	1,817.17 ± 118^a^

Different superscript letters represent statistical difference in line by the Duncan method. Significance level *P* ˂ 0.05. Coefficient of variation < 10. *n* = 6.

It was observed that pequi pulp presented 54.69 mg of gallic acid/100 g, whereas pequi oil obtained 4.45 mg of gallic acid/100 g (Table 1), representing only 8.1% of the concentration of phenolic compounds found in the pulp of pequi.

A total of 24% of the phenolic compounds found in the extracts of pequi pulp and oil are flavonoids (13.29 ± 0.9 mg quercetin/100 g of pulp; 1.1 ± 0.02 mg quercetin/100 g of oil) (Table 1). Among the flavonoids, one class found in pequi was flavanols, which were separated by HPLC-DAD and quantified as total epicatechin, representing 48.02 mg of epicatechin/100 g in pequi and 13.06 mg of epicatechin/100 g in pequi oil, while phenolic acids were determined in a total of 1.27 mg of chlorogenic acid/100 g in pequi oil.

The characterization of the bioactive compounds present in the pequi pulp and oil lipophilic extract was performed by quantification of total carotenoids (Table 1) and identification and quantification of phytosterols and tocopherols (Table 2) present in this fraction. In this study, a comparison of the carotenoid content in the pequi pulp and oil lipophilic extracts was performed, with a 137.6 μg of β-carotene/g of pulp and 549.84 μg of β-carotene/g of pequi oil, demonstrating that the oil has about 4 times more carotenoids than the pequi pulp. In other words, the pequi oil is richer in lipophilic bioactive compounds such as carotenoids, while the pequi pulp is richer than oil in phenolic compounds.

**Table 2:** Phytosterols and tocopherols of pequi oil

Phytosterols	Phytosterol concentration mg/g	Tocopherols	Tocopherol concentration µg/g
Campesterol	1.85	δ+β-tocopherol	12.11
Stigmasterol	0.23	γ-tocopherol	26.24
β-Sitosterol	10.22	α-tocopherol	9.49

b) Results are expressed in average, *n* = 5.

Three phytosterols were identified in pequi pulp oil by HPLC. Based on the retention time of three standards, the concentration of campesterol (1.86 mg/g), stigmasterol (0.23 mg/g), and mainly β-sitosterol (10.22 mg/g) (Table 2) was determined in pequi oil. The same was made for tocopherols determination. Thus, δ + β-tocopherol, γ-tocopherol, and α-tocopherol were identified and quantified in pequi pulp oil, as shown in Table 2. The highest concentrations found in pequi oil were γ-tocopherol (26.24 µg/g sample), followed by δ + β-tocopherol (12.11 µg/g sample) and α-tocopherol (9.49 µg/g sample).

### In vitro *antioxidant capacity*

Table 1 shows the antioxidant capacity of the pulp and oil hydrophilic and lipophilic extracts. The antioxidant capacity of both extracts significantly differed at *P* ˂ 0.05 in all the methods used. For the hydrophilic extract when comparing ABTS, DPPH, FRAP, and ORAC, both oil and pulp had the highest antioxidant capacity by ORAC (*P* > 0.05), and for the lipophilic extract, comparing DPPH and ABTS, the highest value was for the DPPH method (*P* > 0.05).

There was a difference between the antioxidant capacity of the hydrophilic and lipophilic extracts. In the hydrophilic extracts, the pulp presents a higher antioxidant capacity than the oil in all the analyzed methods, while the opposite occurs in the lipophilic extracts, that is, the antioxidant capacity of the oil is higher. This is due to the concentration of bioactive compounds in each type of extract, while the hydrophilic compounds such as flavonoids and flavonols exist in higher quantity in the pulp extract, and the lipophilic compounds such as carotenoids exist in higher quantity in the oil extract.

### Cellular viability assay

**Fig. 1:**
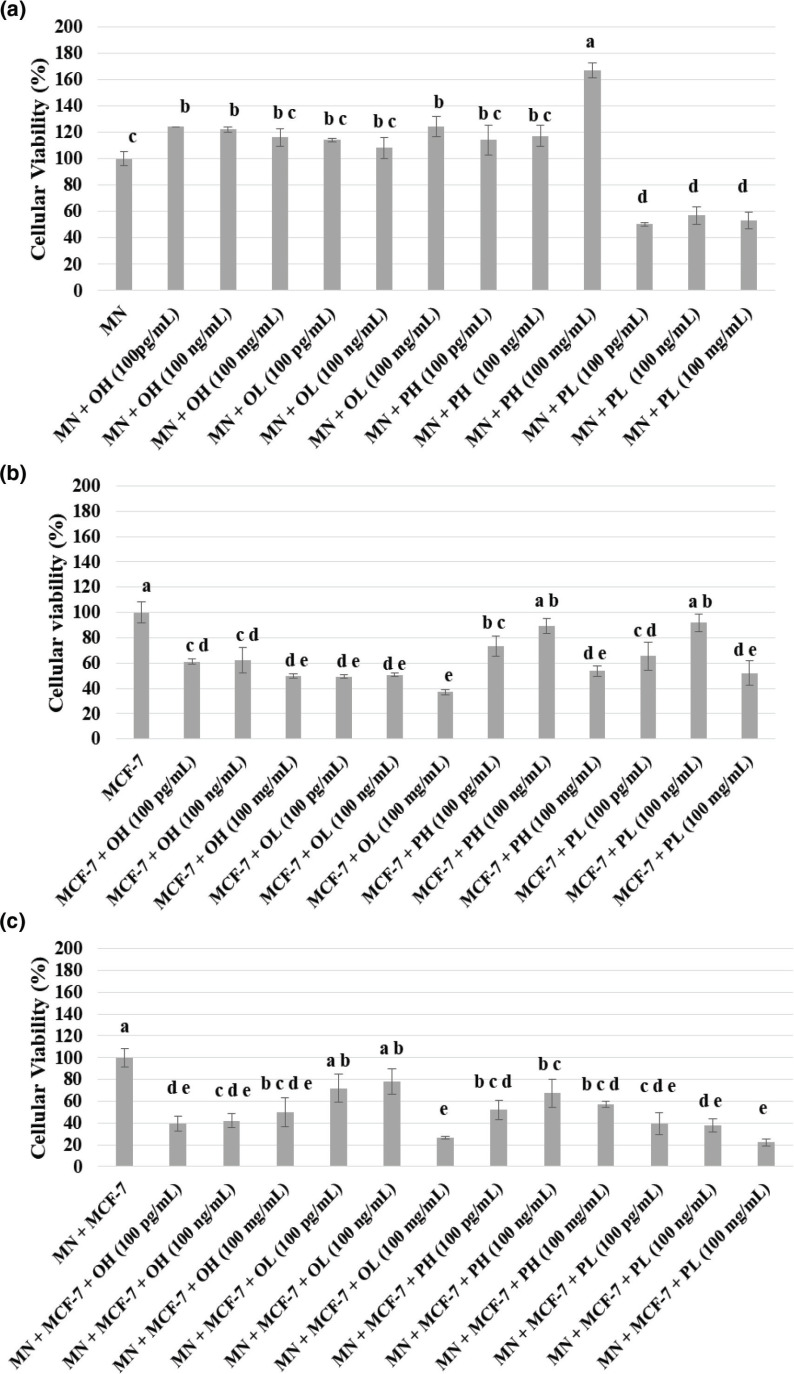
Cellular viability in blood mononuclear cells (a), MCF-7 breast cancer cells (b), and coculture of MN cells with MCF-7 breast cancer cells (c) quantified in the presence of the pequi pulp or oil hydrophilic and lipophilic extracts, treated with different concentration, 100 pg/mL, 100 ng/mL, and 100 mg/mL. MN = blood mononuclear cells; PH = pulp hydrophilic extract; PL = pulp lipophilic extract; OH = oil hydrophilic extract; OL = oil lipophilic extract. Results are expressed in average ± standard deviation, *n* = 4. *Means followed by the same letter do not differ significantly and means followed by different letters differ significantly by the Tukey test (*P* < 0.05).

Figure 1 expresses the results of cytotoxicity of extracts of pequi oil and pulp against human peripheral blood mononuclear cells (Fig. 1a), MCF-7 cells (Fig. 1b), and coculture of both (Fig. 1c).

Analysis of MTT was performed to determine the mitochondrial metabolic rate as an indirect result of cell viability. It was observed that the mononuclear cells submitted to different concentrations of the pequi pulp lipophilic extracts were the only ones that presented a reduction in their viability, varying between 45 and 58% without showing significant difference between the different concentrations tested (*P* ˂ 0.01).

Most of the extracts did not differ statistically from the control (*P* ˂ 0.01). However, when observed the two extracts that presented the highest concentration of bioactive compounds, the pulp hydrophilic extract (highest concentration of phenolic compounds) and the oil lipophilic extract (highest concentration of carotenoids) in concentrations of 100 mg of extract/mL showed an increase in cell viability, reaching approximately 160% in the hydrophilic extract.

Regarding the cell viability of the MCF-7 tumor line and coculture of MN cells and MCF-7 cells, it can be seen that hydrophilic and lipophilic extracts were shown to be cytotoxic in most of the concentrations used, reducing significantly the viability of MCF-7 cells and coculture compared with the control (absence of extracts).

### Cellular-based assays – ex vivo antioxidant activity

**Fig. 2:**
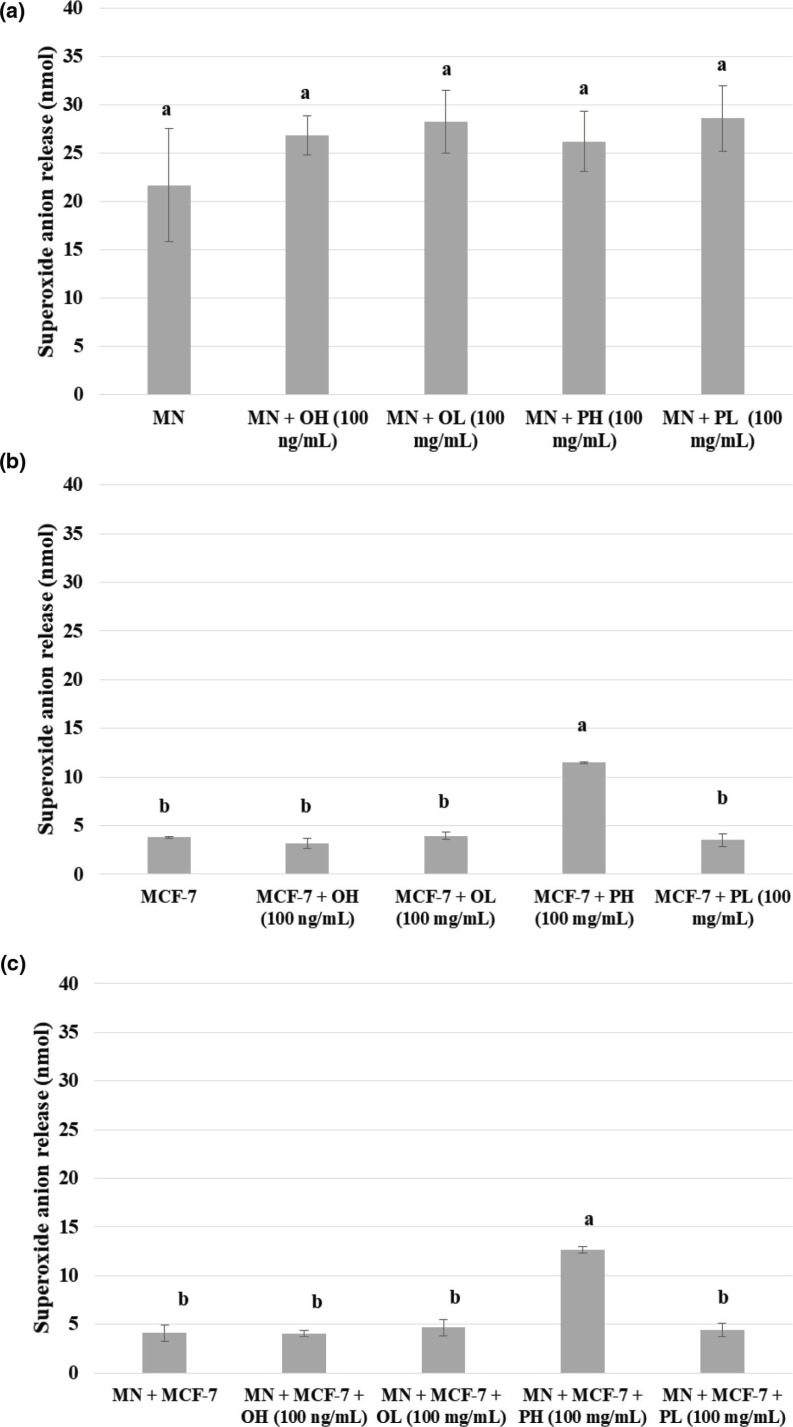
Superoxide anion release (O_2_^●-^) in blood mononuclear cells (a), MCF-7 breast cancer cells (b), and coculture of MN cells with MCF-7 breast cancer cells (c) quantified in the presence of the pequi pulp or oil hydrophilic and lipophilic extracts. MN = blood mononuclear cells; PH = pulp hydrophilic extract; PL = pulp lipophilic extract; OH = oil hydrophilic extract; OL = oil lipophilic extract. Results are expressed in average ± standard deviation, *n* = 5. *Means followed by the same letter do not differ significantly and means followed by different letters differ significantly by the Tukey test (*P* < 0.05).

The analyses of oxidative metabolism were performed by the release of superoxide anion (O_2_^●-^), a reactive oxygen species (ROS) by MN cells, MCF-7 cells, and coculture [MN and MCF-7 cells] in the presence and absence of the pequi extracts (Fig. 2). For this purpose, the concentrations of extracts that showed less cytotoxicity to human peripheral blood mononuclear cells were used.

**Fig. 3:**
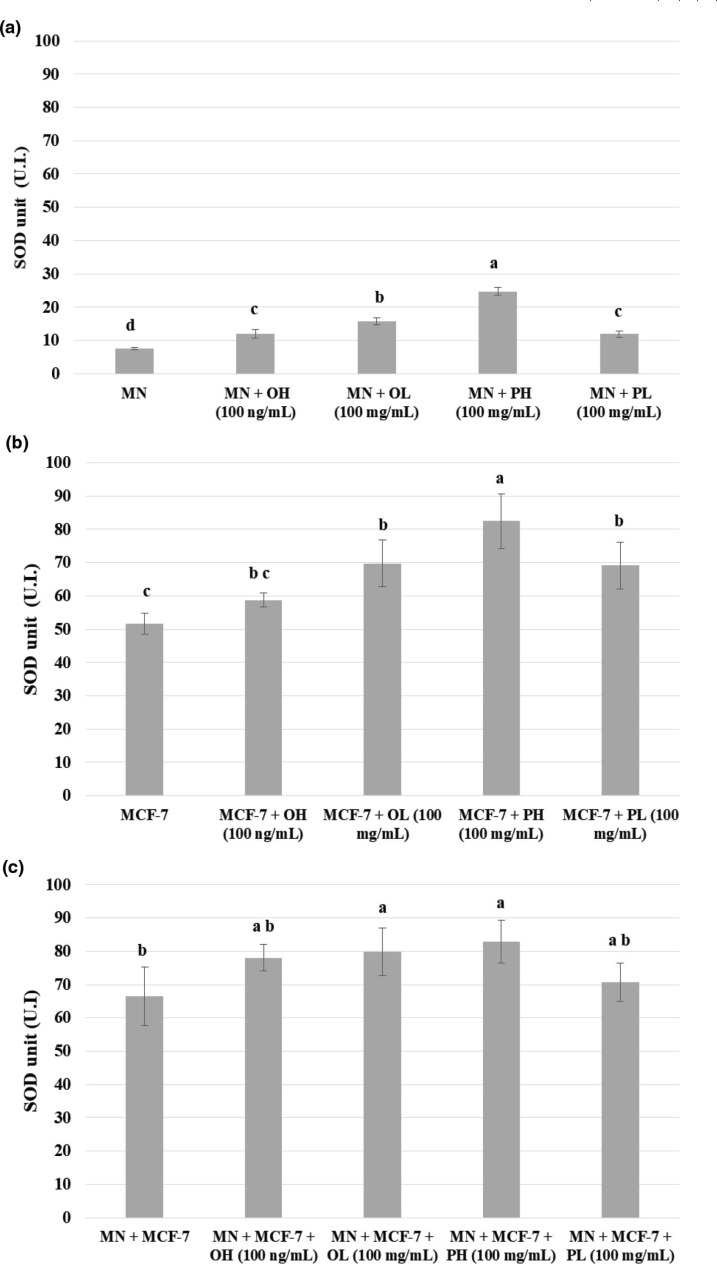
Dismutation value of SOD by blood mononuclear cells (a), MCF-7 breast cancer cells (b) and co-culture of MN cells with MCF-7 breast cancer cells (c) quantified in the presence of the pequi pulp or oil hydrophilic and lipophilic extracts of pequi pulp and oil. MN = blood mononuclear cells; PH = pulp hydrophilic extract; PL = pulp lipophilic extract; OH = oil hydrophilic extract; OL = oil lipophilic extract. Results are expressed in average ± standard deviation, *n* = 3. * Means followed by the same letter do not differ significantly and means followed by different letters differ significantly by the Tukey test (*P* < 0.05).

Figure 3 shows the dismutation levels of the enzyme SOD by MN cells, MCF-7 cells, and coculture [MN and MCF-7 cells] treated with the pequi pulp or oil hydrophilic and lipophilic extracts.

In the present study, no extract influenced the release of O_2_^●−^ by MN cells (*P* ˂ 0.05). The same behavior obtained with MN cells was observed against MCF-7 cells and coculture treated with the extracts, except for the pulp hydrophilic extract at 100 mg/mL, which increased the release of superoxide anion. These results may be related to the fact that this extract in the studied concentration presents the highest flavonoids concentration.

It is observed that the cells treated with all extracts of pequi for 24 h showed a significant increase (*P* ˂ 0.01) in the SOD activity in relation to the control value.

In view of the above, it should be noted that the extracts that had the highest content of bioactive compounds (pulp hydrophilic extract and oil lipophilic extract), at 100 mg/mL, stimulated the human peripheral blood mononuclear cells, resulting in an increase in cell viability. While they were cytotoxic to MCF-7 breast cancer cells and coculture of MN cells and MCF-7 cells, especially the pequi pulp hydrophilic extract at a concentration of 100 mg/mL, which presented the highest immunomodulatory potential for MCF-7 cells and coculture of MN and MCF-7 cells, they induced anion superoxide production and SOD enzyme production in MCF-7 cells, being the main extract with a promising effect to control disorderly tumor growth *in vitro*.

## Discussion

### Bioactive compounds characterization

The higher content of phenolic in pequi pulp when compared with pequi oil is due to the lipophilic character of the oil, which does not have high affinity with the phenolic compounds, since they are water-soluble. According to Machado et al., when working with pequi aqueous and alcohol extracts, the aqueous extract had a higher content of phenolic compounds than the alcohol extract because they diffuse more quickly in water, with a better extraction in a shorter time (6). However, in spite of the low chemical affinity, the oils generally present phenolic compound contents that can vary between 10 and 400 mg/100 g of oil (28).

Other factors that may have contributed to the low content of phenolic compounds in pequi oil compared with the pulp may have been the temperature used for extraction (90°C) and the longtime characteristic of the Goldfish method (approximately 5 h). When observed the study of Torres et al., in which the pequi almond oil was obtained by handmade and cold-pressed processes, the authors verified that the handmade oil presented a phenolic content higher than the cold extraction and suggest that the extraction of the hot oil had a positive influence on the total phenolic concentration in the oil, and the presence of water could also positively contribute to the extraction of these compounds (8). The extraction time may also be a factor contributing to the degradation of the compounds during extraction, reducing the final content in pequi oil due to prolonged exposure to oxygen, polymerization, and interaction of the polyphenols with other constituents of the fruit (29).

Among the phenolic acids already identified in pequi oil, there stand out p-coumaric acid, gallic, p-hydroxybenzoic, caffeic, and ellagic acids (29). These phenolic acids are the most common hydroxycinnamic and hydroxybenzoic acids in wines and are related to antitumor efficacy in breast, ovarian, lung, and oral cancers, due to the induction of cell cycle arrest, inhibition of cell proliferation, reduction of ROS production, induction of apoptosis and autophagy, and the reduction of migration and invasion (30).

Despite the low content of phenolic compounds, other authors (31) when analyzing the phytochemical composition of nine underexplored Brazilian fruits consider that the pequi had the lowest content of phenolic compounds among the fruits analyzed, but in contrast, it presented the highest content of carotenoids, demonstrating that its major compounds have a lipophilic character.

Carotenoids, the liposoluble pigments responsible for color in foods ranging from yellow to red, are the most studied natural pigments in relation to their health effects, through epidemiological, cellular, animal, and even human studies (32). Many studies in the literature have already investigated the carotenoid content in pequi and found that the principal carotenoid present was all-*trans*-zeaxanthin, considering the fruit a potential source of this pigment in both the pulp and its almond (9, 31). In our study, a comparison of the carotenoid content in the pequi pulp and oil lipophilic extracts was performed, and an increase in approximately 300% in the concentration of carotenoids in the oil in relation to the pequi pulp was observed, due to the concentration of these compounds in the lipophilic extract; since there is a reduction of the moisture content in the oil compared with the fruit pulp, the pequi pulp presents 62.9 g/100 g of moisture, while the oil has 4.4 g/100 g of moisture. This reduction in water content also contributes to the conservation of carotenoids, since it reduces enzymatic activity and chemical reactions, both of which cause oxidation and consequent degradation of carotenoids (33). Another factor that may be related to the increased concentration of carotenoids in the oil is due to an increase in bioavailability, which is usually caused by moderate heat treatment (32).

Carotenoids have been shown to inhibit the growth of tumor cells by interfering at different phases of the cell cycle, once they modulate cell cycle arrest by multiple mechanisms in cancer cell, such as G0/G1 phase arrest, G2/M phase arrest, and S phase arrest (34).

To our knowledge, this is the first study reporting the content of phytosterols in pequi pulp oil. Torres et al. reported the need for further research to evaluate the presence of phytochemicals such as phytosterols and tocopherols in oils, among them pequi oil (9). Other study evaluated the content of these compounds in pequi almond oil, finding the same three compounds identified in this study, but differing in relation to the major compound, that was stigmasterol in pequi almond oil (8). Phytosterols, particularly β-sitosterol, have been associated with a lower risk of esophageal cancer and play an important role in inhibiting intestinal cholesterol absorption (12, 35). In this study, pequi oil showed higher amounts of β-sitosterol than other oils such as corn oil (6.908 mg/g), peanut oil (1.889 mg/g), rapeseed oil (4.794 mg/g), and soybean oil (1.894 mg/g) (36).

Regarding the tocopherols, other studies were not found on the composition in pequi pulp oil; only two studies with pequi kernel oil were found, in which the main tocopherol isomer identified in the pequi almond oil (extracted by pressing) was α-tocopherol (79.74–91.49 µg/g), followed by γ-tocopherol (46.62–91.32 µg/g), considered the main tocopherols in vegetable oils and fats and were not found β- and δ-tocopherol (8, 9). A factor that may have contributed to the low tocopherol content in pequi oil may have been the extraction method, which used high temperatures for a long period of time and may have contributed to the degradation of tocopherols.

### In vitro *antioxidant capacity*

The *in vitro* evaluation of the hydrophilic extracts was performed by four methods, ABTS, DPPH, FRAP, and ORAC, while the evaluation of lipophilic extracts was against the DPPH and ABTS assays – methods used as screening to provide an idea of the antioxidant capacity of the extracts, which, when correlated with cellular-based assays such as the determination of SOD (an endogenous antioxidant enzyme) and the formation of superoxide anion in blood mononuclear cells, provide information on the antioxidant activity and immunomodulatory effect of the bioactive compounds in the human body (37).

Regarding the hydrophilic extracts, pequi oil presented a similar ORAC value when compared with other fruit from Brasilian cerrado such as buriti (*Mauritia Flexuosa* L.), which presents 709.0 µmol equivalent Trolox/g of oil (38). The highest results were found in the ORAC assay. This is due to the different reaction mechanisms employed in each method, suggesting that the bioactive compounds present in the pequi hydrophilic extract preferentially act as quenching free radicals by hydrogen donation stabilizing the peroxyl radical by resonance, a fact that can also be verified by the high positive correlation between the content of phenolic compounds of the hydrophilic extract and the value of ORAC and between the content of flavonoids and ORAC.

### Cellular viability assay

The increase in cell viability showed by pulp hydrophilic extract may be related to the high concentration of flavonoids in this extract, which may protect cells from oxidative stress better than carotenoids due to a higher chemo-protection ability of flavonoids (26). Increases in cell viability may occur in the presence of stimuli, such as plant and fruit extracts, due to the antioxidant activity of the compounds present (39).

Regarding the cell viability of the MCF-7 tumor line and coculture of MN cells and MCF-7 cells, the extracts are able to reduce the viability of MCF-7 cells and coculture compared with the control. However, a dose-dependent effect was not observed, as in the study of others where different concentrations of lycopene and β-carotene inhibited the growth of MCF-7 cells (40).

When assessing the cytotoxicity of the lipophilic extracts, the lowest viability percentages were found both for MCF-7 cells (49.2%) and for MN cocultured with MCF-7 cells (22.22%). However, when analyzing the immunomodulatory potential of the pulp hydrophilic extract (100 mg/mL), it was observed that in addition to stimulating the highest viability of MN cells, it also presented cytotoxic profile for tumor cells and coculture. This fact can be attributed to the antioxidant characteristics of the phenolic compounds, presented in the extract, discussed above, demonstrating potential in immunomodulatory activity with relevant perspectives in antitumor activity.

### Cellular-based assays – ex vivo antioxidant activity

The results showed that although the pequi oil and pulp present several bioactive compounds, such as flavonoids, flavanols, carotenoids, phytosterols, and tocopherols, which showed a high antioxidant capacity by the *in vitro* methods, their extracts are not able to decrease the release of O_2_^●−^ by MN cells. Other authors when analyzing ROS scavenging capacity of citharexylum *solanaceum* fruit extracts against HOCl, H_2_O_2_, O_2_, and O_2_^●−^ observed that the extract only showed no activity against the superoxide anion, as this can be rapidly dismutated into H_2_O_2_, due to the enzyme SOD (41).

Experimental studies highlight that exogenous stimulation of ROS production by tumor cells has been associated with the inhibition of cellular proliferation by increasing oxidative stress, resulting in cell death (3). It has recently been shown in ovarian tumor cell culture that flavonoids can induce or inhibit ROS generation according to their structure, hydroxyl number, and concentration (42).

The dismutation levels of the enzyme SOD present a significant role in the elimination of ROS under physiological conditions (43).

The pequi extract has been shown to reduce markers of oxidative stress, increasing the total antioxidant activity by the action of SOD (44). This ability to increase enzymatic activity possibly occurs due to the bioactive compounds present in pequi (9). When analyzing hepatic injury in mice, a study showed that the administration of β-sitosterol could reduce liver tissue damage and restore antioxidant defense, improving the activity of antioxidant enzymes, including SOD (45).

The aforementioned data are consistent with the results of MCF-7 cells, which showed increased SOD levels for all immunomodulatory agents, except for the oil hydrophilic extract.

## Conclusion

The pequi oil presented low content of phenolic compounds in relation to the fruit pulp, but its carotenoid content is high, resulting in high antioxidant activity in both extracts. The major bioactive compounds identified in pequi oil were β-sitosterol and γ-tocopherol. The extracts that had the highest content of bioactive compounds (pulp hydrophilic extract and oil lipophilic extract), at 100 mg/mL, stimulated the human peripheral blood mononuclear cells, resulting in an increase in cell viability. While they were cytotoxic to MCF-7 breast cancer cells and coculture of MN cells and MCF-7 cells, demonstrating immunomodulatory potential mainly for pequi pulp hydrophilic extract, this extract increased the release of superoxide anion and the level of SOD enzyme in MCF-7 cells and coculture.
